# Expenditure and Financial Burden for Stomach Cancer Diagnosis and Treatment in China: A Multicenter Study

**DOI:** 10.3389/fpubh.2020.00310

**Published:** 2020-08-07

**Authors:** Kai Zhang, Jian Yin, Huiyao Huang, Le Wang, Lanwei Guo, Jufang Shi, Min Dai

**Affiliations:** ^1^Department of Cancer Prevention, National Cancer Center/National Clinical Research Center for Cancer/Cancer Hospital, Chinese Academy of Medical Sciences and Peking Union Medical College, Beijing, China; ^2^Department of Cancer Epidemiology, National Cancer Center/National Clinical Research Center for Cancer/Cancer Hospital, Chinese Academy of Medical Sciences and Peking Union Medical College, Beijing, China; ^3^Clinical Trials Center, National Cancer Center/National Clinical Research Center for Cancer/Cancer Hospital, Chinese Academy of Medical Sciences and Peking Union Medical College, Beijing, China; ^4^Office of Cancer Screening, National Cancer Center/National Clinical Research Center for Cancer/Cancer Hospital, Chinese Academy of Medical Sciences and Peking Union Medical College, Beijing, China; ^5^Department of Cancer Epidemiology, Henan Cancer Hospital, Affiliated Cancer Hospital of Zhengzhou University, Zhengzhou, China

**Keywords:** stomach cancer, direct expenditure, indirect expenditure, financial burden, medical expenditure, non-medical expenditure

## Abstract

**Background:** Stomach cancer is a huge threat to the health of Chinese people. However, few studies have looked into the expenditure and financial burden due to stomach cancer in China.

**Methods:** To estimate the direct (medical and non-medical) and indirect expenditure for diagnosis and treatment for stomach cancer patients in China, a multicenter survey was conducted in 37 tertiary hospitals in 13 provinces across China from 2012 to 2014. Each enrolled patient was interviewed through a structured questionnaire. The medical and non-medical expenditure at different clinical stages, the composition of non-medical expenditure, and the time loss for the cancer patient and their family were assessed. All expenditure data were inflated to the 2014 Chinese Yuan [CNY; 1 CNY = 0.163 USA dollar (USD)].

**Results:** A total of 2,401 stomach cancer patients with a mean age of 58.1 ± 11.4 years were included, predominately male. The overall average direct expenditure per patient was estimated to be US $9,899 (medical expenditure 91.2%, non-medical expenditure 8.8%), and the expenditures for stage I, II, III, and IV were $8,648, $9,004, $9,810, and $10,816, respectively; expenditure in stage III and IV was significantly higher than that in stages I and II (*p* < 0.05). One-year out-of-pocket expenditure of a newly diagnosed patient with stomach cancer was $5,368, accounting for 63.8% of their previous-year household income, which led to 79.2% families suffering an unmanageable financial burden. The average loss of time for patients and caregivers was $996.

**Conclusions:** This study indicated that the economic burden of stomach cancer in urban China was onerous. Effective intervention is necessary to reduce the financial burden by reducing the personal payment ratio and increasing the reimbursement ratio.

## Introduction

Stomach cancer incidence and mortality have declined significantly worldwide over the past 4-5 decades, but it remains a global health problem as the fifth most common cancer and the third most common cause of cancer-related deaths worldwide (1). Even today, stomach cancer incidence and mortality remain extremely high in China, which is responsible for 42.6% of the new cases and 45.0% of the death cases in the world (2). In 2015, it was estimated that 679,100 new cases and 498,000 deaths occurred in China (3). Moreover, due to a lack of awareness of physical examination and screening, most patients with stomach cancer have developed to the late stage, missing the optimal treatment time period. Thus, the current status of the stomach cancer epidemic in China dramatically affects people's health and imposes a large financial burden on both the family and society.

Given the growing trend of diagnostic and therapeutic technologies and changes in lifestyles and in people's expectations of the health system, the cost of the healthcare system and the out-of-pocket costs for patients' families are increasingly growing (4). Moreover, many studies have indicated that both cancer patients and their family members suffer a financial burden (4–8). Timely estimation of the costs of care for cancer patients assesses the general status of a population's health under the current healthcare system, which is a vital component of national programs and policies for cancer (9); thus, it enables the development of optimal policies in order to relieve the burden on family and society.

Numerous studies have been carried out on the financial burden of cancer worldwide but rarely on stomach cancer (10–12). Additionally, the majority of them concentrate mainly on the medical expenditure that is documented on hospital information systems; few studies focus on the non-medical expenditure (12–14). Only one multicenter study (five rural and two urban areas) reported direct and indirect expenditure until now (15).

In consideration of the context of the high prevalence of stomach cancer and limited evidence concerning the economic evaluation of stomach cancer in China (12), the study of direct (medical and non-medical) and indirect expenditure of stomach cancer is extremely necessary and meaningful.

In October 2012, the National Health and Family Planning Commission of China initiated the population-based Cancer Screening Program in Urban China (CanSPUC) (16, 17), targeting five types of cancer that are most prevalent in urban areas, i.e., upper digestive tract cancer (stomach cancer and esophageal cancer), lung cancer, breast cancer, liver cancer, and colorectal cancer. Eligible participants are recruited in the communities of the study regions and invited to undertake cancer screening free of charge. As a part of the health economic evaluation research of the CanSPUC, the objective of the study is to estimate both medical and non-medical expenditures overall and for subgroups of stomach cancer patients, to identify the subsequent financial burdens imposed on patient families, and to assess whether the screening strategies involved in the uninterrupted CanSPUC are cost-effective at the current scale or an expanded scale in the future.

## Materials and Methods

### Study Design and Sites

We conducted a multicenter, hospital-based, cross-sectional study in 21 cities from 13 provinces (Shandong, Beijing, Jiangsu, Guangdong, Zhejiang, Hebei, Liaoning, Hunan, Heilongjiang, Henan, Xinjiang, Gansu, and Chongqing) in the east, west, south, and north of China between September 2012 and December 2014, which covered the first 2 years after the CanSPUC startup. A total of 37 tertiary hospitals (23 general hospitals and 14 specialized hospitals) were involved, which is the leading medical hub providing standard and advanced medical care. The details for the 13 provinces concerning population size, gross domestic product (GDP) per capita in 2014 based on the China Statistical Yearbook 2015 (18), number of cities, and general and specialized hospitals are shown in Table S1. The survey was approved by the Institutional Review Board of the Cancer Hospital of the Chinese Academy of Medical Sciences. All patients provided written informed consent.

### Study Subjects

A total of 2,401 stomach cancer patients were expected from the 13 study provinces. Briefly, according to a uniform design scheme, a stratified convenience sampling approach was used for selecting 240 stomach patients from each province. To reach a sufficient power for subgroup analyses, we tried to balance sample sizes among gender (maximum 60% for either gender) and cancer stage (19) (~25% for all clinical stages). All respondents were interviewed face to face using a structured questionnaire at the time of discharge, when most treatment expenses had already been incurred. Family members or caregivers helped with the interview for patients in poor physical condition; otherwise, the patients themselves gave the interview.

### Questionnaire

During the survey process, the subjects were interviewed face to face by trained investigators using a unified questionnaire designed by health economics experts for data collection. The questionnaire included five sections that are introduced briefly as follows (17): (1) demographic and societal information; (2) clinical information; (3) expenditure information for the whole course of illness to date; (4) time loss due to the whole course of clinical visits to date; and (5) quality control. Only if the first four sections were evaluated as of excellent or good reliability would the record be regarded as high quality; otherwise, it was deemed to be low quality, and the subject was excluded from our study. With regard to the inclusion criteria, each patient aged 40–69 years was diagnosed pathologically with primary stomach cancer, primarily underwent treatment at a hospital affiliated with the CanSPUC project, could understand the survey process, and provided informed consent.

### Estimation of Expenditure

The overall expenditure of each patient during the whole course of illness was estimated, which included both medical (amount spent for diagnosis or treatment of stomach cancer) and non-medical (including meals, accommodation, transportation, nutrition, and employee escort fees) expenditures. Medical expenditures were paid in part by the insurers, while non-medical expenditures were paid completely by the patients. Expenditure data indicated the estimates for the whole course of the disease where not specified otherwise. A newly diagnosed course was defined as 2 months before diagnosis and 10 months after diagnosis, which is not completely the same as the commonly used definition (1 year after diagnosis) on the grounds that a great amount of money, in China, is usually spent on diagnosis before the confirmation of the pathological pattern. All patient-paid medical expenditure items and non-medical expenditure of a newly diagnosed course were defined as out-of-pocket expenditure. With the exception of calculating the proportional breakdown of non-medical expenditures, all expenditure data were converted to the 2014 Chinese Yuan (CNY; 1 CNY = 0.163 USD) using the year-specific healthcare consumer price index of China (20).

### Financial Burden

The indicator of the expense-income ratio, equal to the average out-of-pocket expense of a newly diagnosed course divided by the average previous-year household income, was used to objectively reflect the financial burden. Financial catastrophe, using this threshold, occurs with an expense-income ratio equal to or >40% of total household income (21). Furthermore, to further access financial pressure, we asked the patient, “Which of the following accurately describes the financial pressure on your family from your disease?” and offered four response options: “None at all,” “Some, but manageable,” “Heavy,” and “Overwhelming.” “Not at all” and “Some, but manageable” were classified as manageable burdens and the other two responses as unmanageable burdens.

### Quality Control

First, the questionnaire was designed by an experienced expert committee. Second, all well-trained investigators were required to check each questionnaire before completing the survey, and within 2 days, another member of the research staff would subsequently double-check each questionnaire. Third, a data administrator from the National Cancer Center of China would perform a logical test.

### Statistical Analysis

All data were double-entered into EpiData 3.1 software (EpiData Association, Odense, Denmark), and data analysis was performed using SAS 9.2 statistical software (SAS Institute, Cary/NC, USA).

Subgroup analyses of overall expenditure, expense-income ratio, financial pressure, and time loss were conducted. Overall expenditure after logarithm transition, expense-income ratio, and time loss were compared using the two-sample Student's *t*-test for a two-group comparative analysis or ANOVA for more than two groups; the SNK-q test was used for multiple comparisons. The χ^2^-test was used to determine financial pressure. All statistical tests were two-sided, and *p* < 0.05 was considered statistically significant.

## Results

### Patient Characteristics

A total of 2,401 stomach patients with a mean age at diagnosis of 58.1 ± 11.4 years old were finally included. Out of these patients, 607 (25.3%) came from general hospitals and 1,794 (74.7%) from specialized hospitals; 1,677 (69.8%) were men, over twice as many as women (724, 30.2%); the largest numbers of the patients had had primary-school eduction or lower (912, 38.0%) and were farmers (1,066, 44.4%). The mean of previous-year household income and income per patient in the last 5 years were US $8,415 ± $7,863 and $4,476 ± $4,327, respectively. The most common healthcare insurance type was the new rural cooperative medical scheme (1,136, 47.3%), followed by urban employee basic medical insurance (803, 33.4%), and urban resident basic medical insurance (404, 16.8%). The percentages of clinical stage I, II, III, and IV disease were 17.5, 14.4, 27.5, and 38.9%, respectively. More detailed information about the questionnaire results is shown in Table 1.

**Table 1:** Characteristics of patients with stomach cancer.

**Characteristic**	**No. of patients (%)**
**Total**	2,401
**Hospital type**	
General	607 (25.3)
Specialized	1,794 (74.7)
**Age at diagnosis (years)**	
Mean ± SD	58.1 ± 11.4
<45	310 (12.9)
45–54	513 (21.4)
55–64	902 (37.6)
≥65	676 (28.1)
**Gender**	
Men	1,677 (69.8)
Women	724 (30.2)
**Education**	
Primary school or lower	912 (38.0)
Junior high school	784 (32.6)
Senior high school	497 (20.7)
Undergraduate or higher	208 (8.7)
**Occupation**	
Farmer	1,066 (44.4)
Enterprise or company employee/worker	518 (21.6)
Self-employee or unemployed	337 (14.0)
Retiree	227 (9.5)
Public sector employee	211 (8.8)
Other	42 (1.7)
**Previous-year household income (*****n*** **= 2,328)**	
Mean ± SD	51,624 ± 48,237 (CNY) 8,415 ± 7,863 (USD)
Median (P_25_-P_75_)	40,000 (20,000–60,000) (CNY) 6,520 (3,260–9,780) (USD)
<20,000 (CNY)/<3,260 (USD)	421 (18.1)
20,000–39,999 (CNY)/3,260–6,519 (USD)	690 (29.6)
40,000–69,999 (CNY)/6,520–11,409 (USD)	648 (27.8)
≥70,000 (CNY)/≥11,410 (USD)	569 (24.5)
**Income per patient in last 5 years**	
Mean ± SD (*n* = 2,376)	27,458 ± 26,546 (CNY) 4,476 ± 4,327 (USD)
Median (P_25_-P_75_) (*n* = 2,376)	20,000 (10,000–40,000) (CNY) 3,260 (1,630–6,520) (USD)
Number of family members [median (P_25_-P_75_)] (*n* = 2,381)	4 (3–5)
**Healthcare insurance type**	
Urban employee basic medical insurance	803 (33.4)
Urban resident basic medical insurance	404 (16.8)
New rural cooperative medical scheme	1,136 (47.3)
Commercial insurance	14 (0.6)
Self-paid	30 (1.3)
Other	14 (0.6)
**Clinical stage**	
I	420 (17.5)
II	347 (14.4)
III	661 (27.5)
IV	933 (38.9)
Not reported	40 (1.7)
**Pathologic type**	
Adenocarcinoma	1,968 (82.0)
Others	302 (12.6)
Not reported	131 (5.4)
**Number of clinical visits [median (P**_**5**_**-P**_**95**_**)]**	2 (1–5)
**Number of admissions [median (P**_**5**_**-P**_**95**_**)]**	1 (1–5)
**Hospital stay (days) (*****n*** **=** **2,398)**	
Mean ± SD	33 ± 33
Median (P_25_-P_75_)	24 (15–40)
**Quality of the questionnaire**	
High quality	2,267 (94.4)
Low quality	134 (5.6)

*SD, standard deviation; CNY, Chinese Yuan*.

*P_25_-P_75_ percentile 25 to percentile 75*.

*P_5_-P_95_ percentile 5 to percentile 95*.

### Overall, Medical, and Non-medical Expenditures

In 13 provinces, overall expenditure ranged from $6,633 (Chongqing) to $14,352 (Shandong) (Table S1). Spearman correlation analysis indicated that the overall expenditure was not associated with the economy of each site (*r* = 0.154, *p* = 0.616; Table S1). We, therefore, did not use the GDP per capita in the later univariate and multivariate analyses.

When we conducted the univariate analysis to identify subgroups associated with overall expenditure (Table 2), the age at diagnosis (*p* = 0.744) and gender (*p* = 0.177) showed no significant differences; patients in specialized hospitals (*p* < 0.025) or those who were well-educated (undergraduate or higher) (*p* < 0.001) spent more compared with their control groups; farmer patients (*p* < 0.001), those on the new rural cooperative medical scheme (*p* = 0.027), those with a lower household income (*p* = 0.045), or those who were diagnosed with adenocarcinoma (*p* < 0.001) spent less. Multivariate analysis was conducted and showed that patients who were diagnosed at stages IV (*p* < 0.001) and III (*p* < 0.008), with undergraduate or higher eduction (*p* < 0.001) or were retired (*p* < 0.003) and public-sector employees (*p* < 0.003) spent more compared with their control groups. Tables 3, 4 showed the expenditure details.

**Table 2:** Univariate analysis of expenditure for diagnosis and treatment of 2,401 patients with stomach cancer.

**Variable**	**Medical expenditure (USD)**	**Non-medical expenditure (USD)**	**Overall expenditure**	
			**Value (USD)**	***p*^a^**
**Total**	9,029	871	9,899	–
**Hospital type**				
General	8,401	752	9,153	**0.025**
Specialized	9,241	911	10,152	
**Age at diagnosis (years)**				
<45	9,039	1,001	10,040	0.744
45–54	9,066	934	10,000	
55–64	9,039	859	9,897	
≥65	8,982	778	9,761	
**Gender**				
Men	8,963	858	9,822	0.177
Women	9,180	899	10,079	
**Education**				
Primary school or lower	8,490	720	9,209	**<0.001**
Junior high school	9,007	870	9,877	
Senior high school	8,994	934	9,928	
Undergraduate or higher	11,556	1,383	12,938	
**Occupation**				
Farmer	8,540	777	9,317	**<0.001**
Enterprise or company employee/worker	9,113	910	10,023	
Self-employee or unemployed	8,587	827	9,413	
Retiree	11,101	1,350	12,451	
Public sector employee	10,018	811	10,829	
Other	7,768	822	8,590	
**Healthcare insurance type**				
Urban employee basic medical insurance	9,507	986	10,494	**0.027**
Urban resident basic medical insurance	8,874	925	9,799	
New rural cooperative medical scheme	8,714	755	9,468	
Commercial insurance	10,046	921	10,967	
Self-paid	9,322	956	10,278	
Others	10,279	1,703	11,982	
**Previous-year household income**				
<20,000 (CNY)/<3,260 (USD)	8,324	859	9,183	**0.045**
20,000–39,999 (CNY)/3,260–6,519 (USD)	8,933	854	9,788	
40,000–69,999 (CNY)/6,520–11,409 (USD)	9,629	823	10,452	
≥70,000 (CNY)/≥11,410 (USD)	9,130	945	10,075	
**Pathologic type**				
Adenocarcinoma	8,977	853	9,830	**<0.001**
Others	9,298	919	10,217	

*CNY, Chinese Yuan; USD, USA dollar*.

a *Two-sample Student's t-test or ANOVA after logarithm transition for two groups or greater than two groups in the comparative analysis, respectively*.

*The bold values are considered statistically significant (p < 0.05)*.

**Table 3:** Multivariate analysis of overall expenditure for diagnosis and treatment of 2,401 patients with stomach cancer.

**Characteristic**	**Estimate (95%CI)**	***p***
**Intercept**	10.75	**<0.001**
**Hospital type**		
General	Reference	
Specialized	0.06 (−0.01 to 0.14)	0.077
**Age at diagnosis (years)**		
<45	−0.04 (−0.14 to 0.07)	0.500
45–54	0.01 (−0.08 to 0.10)	0.758
55–64	0.02 (−0.05 to 0.10)	0.586
≥65	Reference	
**Gender**		
Men	−0.06 (−0.12 to 0.01)	0.086
Women	Reference	
**Education**		
Primary school or lower	Reference	
Junior high school	0.06 (−0.01 to 0.14)	0.083
Senior high school	0.05 (−0.04 to 0.14)	0.301
Undergraduate or higher	0.26 (0.13 to 0.40)	** <0.001**
**Occupation**		
Farmer	Reference	
Enterprise or company employee/worker	0.10 (−0.01 to 0.22)	0.072
Self-employee or unemployed	−0.00 (−0.11 to 0.10)	0.931
Retiree	0.23 (0.08 to 0.38)	**0.003**
Public sector employee	0.21 (0.07 to 0.34)	**0.003**
Other	−0.01 (−0.25 to 0.22)	0.914
**Healthcare insurance type**		
Urban employee basic medical insurance	−0.10 (−0.21 to 0.01)	0.071
Urban resident basic medical insurance	−0.02 (−0.11 to 0.08)	0.746
New rural cooperative medical scheme	Reference	
Commercial insurance	0.10 (−032 to 0.52)	0.641
Self-paid	0.12 (−0.18 to 0.41)	0.443
Others	−0.02 (−0.43 to 0.39)	0.926
**Previous-year household income**		
<20,000 (CNY)/ <3,260 (USD)	Reference	
20,000–39,999 (CNY)/3,260–6,519 (USD)	0.03 (−0.05 to 0.12)	0.446
40,000–69,999 (CNY)/6,520–11,409 (USD)	0.07 (−0.03 to 0.16)	0.157
≥70,000 (CNY)/≥11,410 (USD)	0.03 (−0.07 to 0.12)	0.610
**Clinical stage**		
I	Reference	
II	0.03 (−0.07 to 0.14)	0.542
III	0.12 (0.03 to 0.21)	**0.008**
IV	0.21 (0.12 to 0.29)	** <0.001**
**Pathologic type**		
Adenocarcinoma	0.00 (−0.08 to 0.09)	0.931
Others	Reference	

*CNY, Chinese Yuan; USD, USA dollar; CI, confidence interval*.

*The bold values are considered statistically significant (p < 0.05)*.

**Figure 1:**
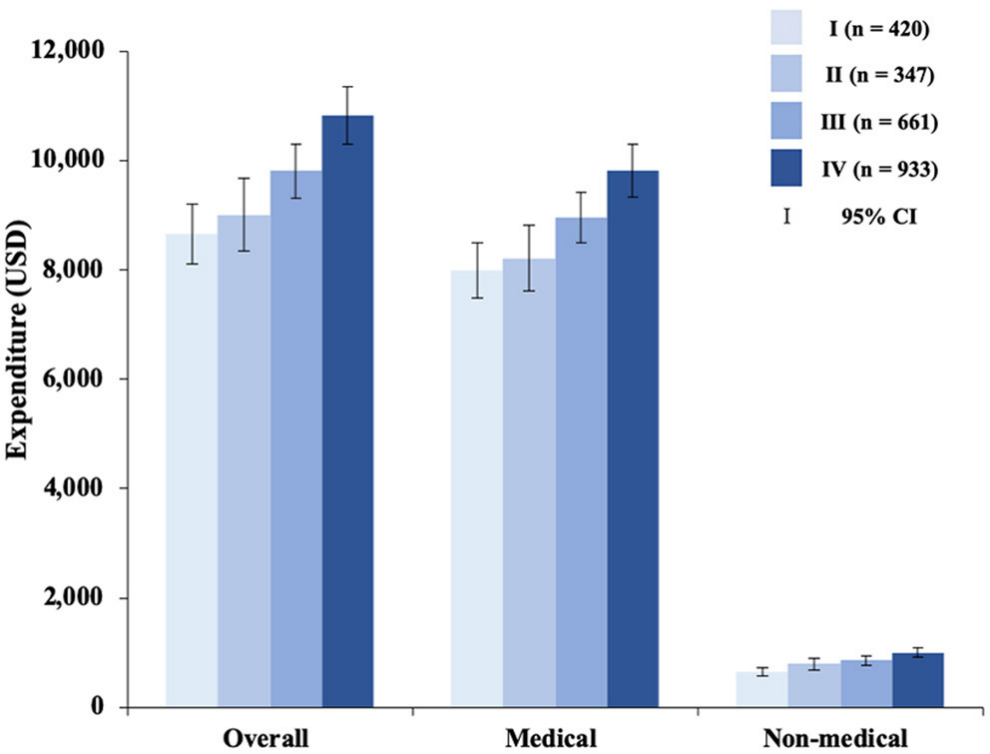
Medical and non-medical expenditure for stomach cancer diagnosis and treatment by clinical stage. Of the 2,361 patients included, 40 supplied no information on clinical stage. USD, USA dollar; CI, confidence interval.

Overall the mean expenditure of each stomach cancer patient was $9,899, with 91.2% ($9,029) being accounted for by medical expenditure and 8.8% ($871) by non-medical expenditure (Table 2). The overall, medical, and non-medical expenditures all showed a dramatic rise with progression of the disease (all *p* < 0.001; Figure 1).

When we made multiple comparisons for clinical stages in terms of overall, medical, and non-medical expenditures, stages I and III, I–IV, II–III, and II–IV diseases showed significant differences in both overall and medical expenditure (*p* < 0.05); stages I–II, I–III, I–IV, and II–IV showed significant differences in non-medical expenditure (*p* < 0.05). Overall expenditure ranged from $8,648 (95% CI $8,098–$9,198) for stage I disease, to $9,810 CNY (95% CI $9,318–$10,303) for stage III disease, with a 13.4% growth rate, to $10,816 CNY (95% CI $10,290–$11,342) for stage IV disease, with a 25.1% growth rate; medical expenditure ranged from $7,992 (95% CI $7,480–$8,505) for stage I disease, to $8,957 (95% CI $8,505–$9,409) for stage III disease, with 12.1% a growth rate, to $9,816 (95% CI $9,336–$10,296) for stage IV disease, with a 22.8% growth rate; non-medical expenditure ranged from $656 (95% CI $583–$729) for stage I disease, to $854 (95% CI $775–$932) for stage III disease, with 30.2% a growth rate, to $1,000 (95% CI $919–$1,081) for stage IV disease, with a 52.5% growth rate (Figure 1).

**Figure 2:**
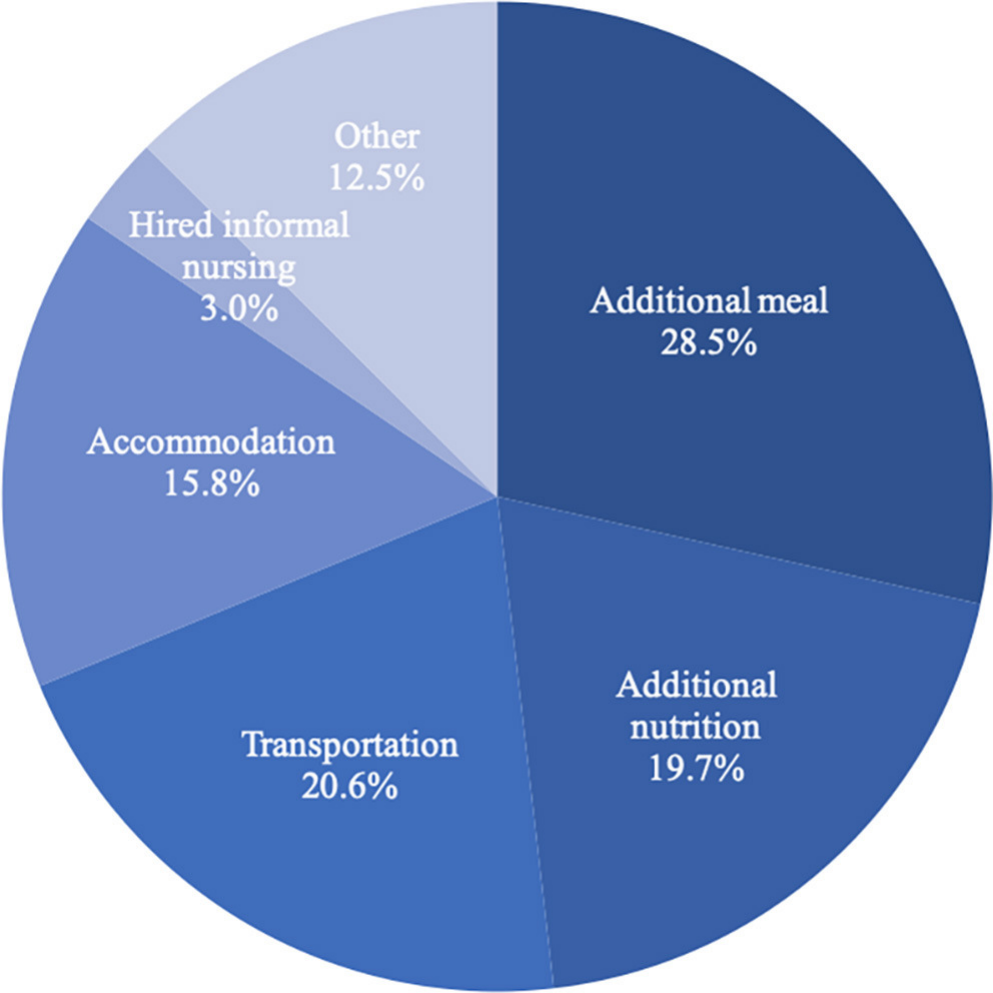
The proportional breakdown of non-medical expenditure for stomach cancer diagnosis and treatment.

In non-medical expenditure, additional meals accounted for the largest proportion ($248, 28.5%), followed by transportation ($179, 20.6%) and additional nutrition ($171, 19.7%). Detailed information about non-medical expenditure is shown in Figure 2.

### Financial Burden

The overall expenditure of a newly diagnosed illness course was $9,155, which was responsible for 92.5% ($9,899) of the total expenditure of the whole course of illness. The self-reported predicted reimbursement ratio was 43.0%; out-of-pocket expenditure ($5,368) accounted for 63.8% of the previous-year household income ($8,415). Of all patient families, 79.2% of the families felt an unmanageable burden (29.7% heavy, 49.5% overwhelming), 16.1% felt some burden but considered it manageable, and only 4.7% felt no burden at all.

Patients who were diagnosed at a younger age (≤54 years) felt more of an unmanageable burden than those diagnosed at an older age (*p* < 0.001). The expense-income ratio for patients with poor education (primary school or lower) was 0.76; the patients feeling an unmanageable burden accounted for 84.1% (28.7% heavy, 55.4% overwhelming), which was higher than for well-educated patients (*p* < 0.001). The expense-income ratio for patients who were farmers was 0.89, which was the highest in all occupations; 88.2% the farmer patients considered the burden unmanageable, and they felt a higher financial burden than any other occupation group (*p* < 0.001).

Patients who had the new rural cooperative medical scheme insurance spent 88.0% of their household income. However, 90% (23.3% heavy, 66.7% overwhelming) self-pay patients experiencing unmanageable financial pressure, which is higher than other healthcare insurance types, although the out-of-pocket expenditure of self-pay patients accounted for only 61% of the previous-year household income. Just as we expected, patients with lower household income (<20,000 CNY/<3,260 USD) experienced dramatic financial pressure, and they had spent more than 3.5 times the previous-year household income, making 95.5% (18.8% heavy, 76.7% overwhelming) of these families unable to afford treatment. We still found that families of stage I stomach cancer patients suffered the lowest pressure (76.9%), but it was not small. Financial pressure did not show significant differences with Hospital type (*p* = 0.056) and gender (*p* = 0.995). More information about the financial burden of overall expenditure is shown in Table 4.

**Table 4:** Financial effect of overall expenditure on stomach cancer patient's family.

**Characteristic**	**Expenditure of a newly diagnosed disease course^a^ (USD)**	**Self-reported predicted reimbursement ratio (%) (*n* = 2,380)**	**Out-of-pocket expenditure^b^ (USD) (A)**	**Previous-year household income (USD) (B)**	**Expense-income ratio (A/B) (*n* = 2,328)**	**Self-reported financial pressure (%) (*****n*** **=** **2,392)**				***p^c^***
						**None at all**	**Some, but manageable**	**Heavy**	**Overwhelming**	
**Total**	9,155	43.0	5,368	8,415	0.64	4.7	16.1	29.7	49.5	**–**
**Hospital type**										
General	8,236	44.4	4,838	10,246	0.47	6.6	16.7	29.2	47.4	0.056
Specialized	9,465	42.5	5,549	7,792	0.71	4.0	15.9	29.9	50.2	
**Age at diagnosis (years)**										
<45	9,163	44.3	5,483	10,079	0.54	2.9	14.6	25.6	57.0	** <0.001**
45–54	9,244	39.2	5,729	8,469	0.68	3.1	14.0	27.7	55.2	
55–64	9,127	42.2	5,364	7,899	0.68	4.2	14.9	30.3	50.6	
≥65	9,120	46.2	5,043	8,286	0.61	7.3	19.9	32.4	40.3	
**Gender**										
Men	9,124	42.7	5,365	8,140	0.66	4.7	16.0	29.8	49.5	0.995
Women	9,255	43.6	5,373	9,048	0.59	4.6	16.3	29.6	49.5	
**Education**										
Primary school or lower	8,553	38.4	5,316	7,033	0.76	2.9	13.0	28.7	55.4	** <0.001**
Junior high school	9,171	41.8	5,513	8,061	0.68	4.0	15.5	29.3	51.2	
Senior high school	9,055	47.2	5,123	9,370	0.55	7.4	19.3	30.4	42.9	
Undergraduate or higher	11,968	57.4	5,641	13,359	0.42	8.7	24.3	34.0	33.0	
**Occupation**										
Farmer	8,755	35.2	5,828	6,566	0.89	1.7	10.2	26.4	61.8	** <0.001**
Enterprise or company employee/worker	9,181	52.5	4,801	9,856	0.49	7.2	17.4	29.3	46.1	
Self-employee or unemployed	8,425	33.8	5,531	8,426	0.66	3.0	16.9	36.2	43.9	
Retiree	11,763	59.4	5,339	11,440	0.47	10.2	23.6	30.7	35.6	
Public sector employee	9,721	59.2	4,158	10,580	0.39	9.0	33.3	32.4	25.2	
Others	7,893	27.3	5,554	9,103	0.61	11.9	16.7	50.0	21.4	
**Healthcare insurance type**										
Urban employee basic medical insurance	9,672	57.1	4,485	10,440	0.43	9.0	22.4	31.3	37.4	** <0.001**
Urban resident basic medical insurance	8,950	47.1	4,859	7,905	0.61	4.5	19.6	30.9	45.0	
New rural cooperative medical scheme	8,867	32.6	6,083	6,933	0.88	1.6	10.4	28.6	59.5	
Commercial insurance	10,573	36.0	6,886	16,250	0.42	7.1	7.1	28.6	57.1	
Self-paid	8,039	0.0	8,039	13,134	0.61	0.0	10.0	23.3	66.7	
Others	9,617	60.1	5,074	8,739	0.58	23.1	46.2	15.4	15.4	
**Previous-year household income**										
<20,000 (CNY)/ <3,260 (USD)	8,623	35.6	5,744	1,601	3.59	1.4	3.1	18.8	76.7	** <0.001**
20,000–39,999 (CNY)/3,260–6,519 (USD)	9,047	37.7	5,791	4,230	1.37	2.8	10.5	29.9	56.9	
**Clinical stage**										
40,000–69,999 (CNY)/6,520–11,409 (USD)	9,721	44.7	5,420	8,111	0.67	4.3	21.7	34.1	39.9	
≥70,000 (CNY)/≥11,410 (USD)	9,165	53.6	4,509	18,878	0.24	9.4	26.7	34.3	29.7	
I	8,084	38.9	5,063	7,814	0.65	6.0	17.1	35.5	41.4	**0.002**
II	8,522	40.2	5,146	7,659	0.67	5.8	12.5	27.0	54.7	
III	9,307	43.0	5,543	7,897	0.70	2.6	17.0	28.8	51.6	
IV	9,706	46.0	5,420	9,203	0.59	4.9	16.8	28.9	49.4	
**Pathologic type**										
Adenocarcinoma	9,113	43.4	5,303	8,561	0.62	4.4	16.2	28.7	50.7	**0.002**
Others	9,264	44.2	5,352	8,458	0.63	7.0	18.5	34.9	39.6	

*CNY, Chinese Yuan; USD, USA dollar*.

a *Two months before and 10 months after diagnosis*.

b *Out-of-pocket expenditure = [∑ (1 – self-reported predicted reimbursement ratio) × medical expenditure of a newly diagnosed course + non-medical expenditure of a newly diagnosed course]/n; n refers to the sample size*.

c *We classified “none at all” and “some, but manageable” as manageable burdens and the other two responses as unmanageable burdens. The Chi-square test was used for subgroup comparison*.

*The bold values are considered statistically significant (p < 0.05)*.

### Time Loss

Mean overall time loss totaled 88.1 person-days, including 48.8 person-days (55.4%) for patients and 39.3 person-days (44.6%) for caregivers. The average value of loss of time was $996.

Patients whose previous-year household income was <20,000 CNY (<3,260 USD) (*p* < 0.001) or those who were diagnosed with adenocarcinoma (*p* = 0.018) suffered relatively more time loss than patients who had higher income or those who were diagnosed with other pathologic types; on the other hand, patients who were diagnosed with stage I–II disease (*p* < 0.021) suffered less than their corresponding control groups, whereas stratified by the other subgroups hospital type (*p* = 0.808), age at diagnosis (*p* = 0.251), gender (*p* = 0.393), education (*p* = 0.588), occupation (*p* = 0.514), or healthcare insurance type (*p* = 0.085), there was no difference (Table 5).

**Table 5:** Time loss due to stomach cancer diagnosis and treatment.

**Characteristic**	**Time loss (person-days)**			***p*^a^**
	**Overall (*n* = 2,303)**	**Patients (*n* = 2,303)**	**Caregivers (*n* = 2,303)**	
**Total**	88.1	48.8	39.3	**–**
**Hospital type**				
General	82.2	43.5	38.7	0.808
Specialized	90.1	50.6	39.5	
**Age at diagnosis (years)**				
<45	88.8	51.0	37.8	0.251
45–54	86.3	46.8	39.5	
55–64	93.0	52.6	40.4	
≥65	82.5	44.1	38.4	
**Gender**				
Men	87.8	48.4	39.4	0.393
Women	88.8	49.7	39.1	
**Education**				
Primary school or lower	87.0	48.8	38.2	0.588
Junior high school	91.1	50.5	40.6	
Senior high school	84.2	45.8	38.4	
Undergraduate or higher	91.1	49.2	41.9	
**Occupation**				
Farmer	89.4	50.6	38.8	0.514
Enterprise or company employee/worker	86.6	46.4	40.2	
Self-employee or unemployed	82.2	45.4	36.8	
Retiree	92.8	50.5	42.3	
Public sector employee	87.2	48.2	38.9	
Others	100.2	53.2	47.0	
**Healthcare insurance type**				
Urban employee basic medical insurance	86.9	47.0	39.9	0.085
Urban resident basic medical insurance	95.8	52.7	43.1	
New rural cooperative medical scheme	87.1	49.2	37.9	
Commercial insurance	78.0	42.5	35.5	
Self-paid	57.3	30.5	26.8	
Others	93.3	48.5	44.8	
**Previous-year household income**				
<20,000 (CNY)/<3,260 (USD)	98.1	54.7	43.4	** <0.001**
20,000–39,999 (CNY)/3,260–6,519 (USD)	94.2	52.2	42.0	
40,000–69,999 (CNY)/6,520–11,409 (USD)	86.1	47.8	38.3	
≥70,000 (CNY)/≥11,410 (USD)	76.1	42.0	34.2	
**Clinical stage**				
I	77.1	43.1	34.0	**0.021**
II	79.2	42.3	36.9	
III	86.5	47.7	38.8	
IV	97.5	54.8	42.7	
**Pathologic type**				
Adenocarcinoma	88.6	49.4	39.2	**0.018**
Others	79.4	43.5	35.9	

*CNY: Chinese Yuan, USD: USA dollar*.

a *Two-sample Student's t-test or ANOVA after logarithm transition for two groups or greater than two groups in the comparative analysis, respectively*.

*The bold values are considered statistically significant (p < 0.05)*.

## Discussion

In China, stomach cancer is the second most commonly diagnosed cancer in men and the third most common in women. Unlike other countries, the incidence and mortality rate continue to increase or are stable. Almost half of the new cases in 2012 occurred in China (2). Moreover, the hospitalization expense of treatment for stomach cancer increased by 49.5% from 2011 to 2015 after adjusting for purchasing power in China (22), indicating that the financial burden is heavy. Given that there is a lack of evidence on direct (medical and non-medical expenditure) and indirect expenditure in China, our investigation is therefore crucial for the medical security system and policy-making.

The study first reported multicenter data from more than 10 provinces in China concerning the direct and indirect medical expenditure of patients with stomach cancer. A total of 2,041 stomach patients from 13 provinces were included, most of whom were diagnosed and treated in specialized hospitals (74.7%). The number of men was more than double that of women, which was almost the same as the study by the National Central Cancer Registry: estimated new male and female cancer patients in China was 70.3 and 29.7%, respectively (2).

The average overall expenditure per patient was calculated to be $9,899 (medical expenditure $9,029, non-medical expenditure $871), and the overall expenditure was not associated with the economy of each province on the basis of Spearman correlation analysis (*r* = 0.154, *p* = 0.616). The amount is far higher than that reported by Zhao et al. (23) in Anhui province, China (overall expenditure $2,586, medical expenditure $2,384, and non-medical expenditure $201), those reported by Xu et al. (24) in Xinjiang province, China ($794 medical expenditure, accounting for 92.4%; $65 non-medical expenditure, accounting for 7.6%), and those reported by Sun et al. (14) from 13 provinces in China (medical expenditure $7,050, but non-medical expenditure not included), but lower than the study by Li et al. in Taiwan province ($10,780 for initial care of per patient with stomach cancer) (25). Looking at foreign countries, total medical costs for a patient with stomach cancer were $6,500 in Iran in 2015, which was lower than found in our study (4).

In our multicenter study, medical expenditure for late-stage cancer (III and IV) was higher than for care for early-stage cancer (I and II), which was consistent with previous studies (4, 26). It is well-known that late-stage patients have longer hospitalizations and more expensive therapy, such as radiotherapy, chemotherapy, and targeted therapy, which are almost never used for those patients in the early stage, and this is linked with higher cost. On the other hand, the early-stage patients tend to undergo surgery, the cost of which is less. This indicated that the high prevalence and diagnosis of disease at advanced stages of disease impose great costs on the patients and the health system. However, a multicenter study in China showed the opposite result in urban areas (15). The possible cause of this discrepancy is that in their study, the cases mainly came from cites with low GDP, where advanced patients are likely to forego expensive treatment due to their low incomes.

Although we attempted to balance stage-specific cases, only 17.5% of all cases were stage I, while 66.4% (27.5% in stage III and 38.9 in stage IV) were late-stage, which reflects the epidemic status of stomach cancer where most detected patients are late stage and there is a lack early diagnosis and treatment. Moreover, both in terms of medical and non-medical expenditures and of financial burden and time loss, late-stage patients paid more. Therefore, early diagnosis through screening and selecting an appropriate treatment method might substantially ameliorate the economic burden of the disease.

In addition, non-medical expenditure for the diagnosis and treatment of stomach cancer constituted a significant component, reaching 8.8% of the overall expenditure (additional meals, 28.5%; transportation, 20.6%; nutrition, 19.7%), which was higher than reported in the previous relevant study in China (only 5.5%) (24). In detail, transportation, nutrition and other non-medical expenditures accounted for 40.3, 17.5, and 42.2%, respectively. Moreover, the non-medical expenditure for late-stage cancer (III and IV) was also higher than that for early-stage cancer (I and II), possibly because patients with late-stage cancer need to go to and from the hospital more times and also need more nutrients.

Concerning financial burden, patients spent 63.8% of their household income for 1 year of stomach cancer diagnosis and treatment, and 79.2% of the families perceived the financial burden as unmanageable. We used the threshold proposed by Xu et al. that financial catastrophe occurs with health care payments at or exceeding 40% of a household's income each year (21); this indicates that the expenditure of our patients was catastrophic. Our result was the same as that of the study by Li et al. (27), which found that out-of-pocket cost per hospitalization accounted for 79% of per capita disposable income in Hua country in 2011. In the United States, in contrast, 25.0% of beneficiaries with the largest burden spent at least 29.9% of their annual income on healthcare (28). This indicated that the economic burden imposed by stomach cancer was relatively high for individual patients' families in China, especially for low-income families.

The loss of time of patients and their families due to the lack of a normal work schedule as a result of illness and treatment, including spending traveling to and from care, waiting for appointments, and so forth, was considered as an indirect economic impact. Time loss therefore represents an essential component of the burden of illness for patients and caregivers. However, few studies have estimated the time costs of patients with stomach cancer. In this study, the average loss of time was estimated at 88.1 days, equaling $996, which is 12.9% of per capita GDP, which crudely converted to a minimum wage loss of $254 in Beijing in 2014. In the USA, data from Surveillance, Epidemiology, and End Results linked to Medicare claims (SEER-Medicare) showed that the mean patient time cost was $5,348 (351.3 days, 12.1% GDP) in initial phase (29). Although the cost is higher than in our study, the proportion of GDP was lower, suggesting that our burden of stomach cancer was heavier. Furthermore, patients with late-stage cancer spent significantly more through time loss than those in the earlystage, not only in patients but also in caregivers. Thus, it is essential to reduce indirect costs through early diagnosis and treatment.

The study had several limitations. First, we only counted the expenditure data within the study hospitals, and it is possible that some patients had received diagnosis and therapy from other hospitals in China, indicating that we probably underestimated the economic burden. Future study needs to overcome this. Second, the retrospective nature of the questionnaire with face-to-face interview may contribute to recall bias. Third, the majority of patients being from specialized hospitals may mean that there is selection bias; nevertheless, considering that most cancer patients prefer to choose and transfer specialized hospitals, the bias can be regarded as tiny.

## Conclusion

In conclusion, our study has directive significance in the development of optimal policies of the healthcare system. This study indicated that direct and indirect expenditures for stomach cancer in China were onerous. It is essential that effective measures be taken in order to reduce the financial burden by decreasing the personal payment ratio and increasing the reimbursement ratio. Cancer screening is cost-effective due to the fact that early detection and therapy of stomach cancer can dramatically reduce costs. Studies on the financial burden of stomach cancer in China are still rare, especially multicenter studies, and more research is sorely needed in the future.

## Data Availability Statement

All datasets generated for this study are included in the article/Supplementary Material.

## Ethics Statement

The studies involving human participants were reviewed and approved by this survey was approved by the Institutional Review Board of the Cancer Hospital of the Chinese Academy of Medical Sciences. The patients/participants provided their written informed consent to participate in this study.

## Author Contributions

KZ, JY, JS, and MD: study concepts and study design. HH, JY, LW, and LG: data acquisition, analysis, interpretation, and statistical analysis. JY and KZ: manuscript preparation and editing. KZ, HH, JS, and MD: manuscript review. All authors read and approved the final manuscript.

## Conflict of Interest

The authors declare that the research was conducted in the absence of any commercial or financial relationships that could be construed as a potential conflict of interest.
