# Supplementary material for: Expenditure and Financial Burden for Stomach Cancer Diagnosis and Treatment in China: A Multicenter Study
Source: Front Public Health. 2020 Aug 7;8:310. doi: 10.3389/fpubh.2020.00310 (PMC7426494; doi:10.3389/fpubh.2020.00310)
Supplement: Supplementary file 1 [file Table_1.DOCX]

**Table S1 Summary information and overall expenditure for diagnosis and treatment of patients with stomach cancer in 13 study provinces in China**

| **Province** | **General information** | |  | **Specific information on cites and hospitals involved** | | | | **Overall**  **Expenditure (USD)** |
| --- | --- | --- | --- | --- | --- | --- | --- | --- |
|  | **Population size in 2014^a^ (×10,000)** | **GDP per capita in 2014^a^ (USD)** |  | **Number of cities** | **Total number of hospitals** | **Number of general hospitals** | **Number of specialized hospitals** |  |
| **Shandong** | 9789 | 9923 |  | 1 (Jinan) | 1 | 0 | 1 | 14352 |
| **Beijing** | 2152 | 16299 |  | 1 (Beijing) | 3 | 1 | 2 | 12479 |
| **Xinjiang** | 2298 | 6626 |  | 1 (Urumchi) | 2 | 0 | 2 | 12441 |
| **Hunan** | 6737 | 6564 |  | 1 Changsha) | 6 | 5 | 1 | 11940 |
| **Guangdong** | 10724 | 9542 |  | 5 (Guangzhou, Shenzhen, Zhongshan, Dongguan, and Foshan) | 2 | 1 | 1 | 9997 |
| **Zhejiang** | 5508 | 11899 |  | 2 (Hangzhou, Ningbo) | 3 | 3 | 0 | 9963 |
| **Heilongjiang** | 3833 | 6394 |  | 2 (Harbin, Daqing) | 1 | 1 | 0 | 8972 |
| **Gansu** | 2591 | 4309 |  | 2 (Lanzhou, Jinchang) | 1 | 0 | 1 | 8490 |
| **Henan** | 9436 | 6043 |  | 1 (Zhengzhou) | 6 | 5 | 1 | 8477 |
| **Hebei** | 7384 | 6517 |  | 1 (Tangshan) | 1 | 0 | 1 | 7423 |
| **Jiangsu** | 7960 | 13345 |  | 2 (Nantong, Xuzhou) | 1 | 0 | 1 | 7330 |
| **Liaoning** | 4391 | 10628 |  | 1 (Tieling) | 9 | 7 | 2 | 6899 |
| **Chongqing** | 2991 | 7800 |  | 1 (Chongqing) | 1 | 0 | 1 | 6633 |
| **National total** | 136,782 | 7604 |  | 21 | 37 | 23 | 14 | 9899^b^ |

USD, USA dollar; GDP, gross domestic product

^a^ Based on China Statistical Yearbook 2015. http://www.stats.gov.cn/tjsj/ndsj/2015/indexch.htm [17]

^b^ The average overall expenditure for colorectal cancer diagnosis and treatment based on data from the 13 study sites in China
